# Enhanced Second Harmonic Generation by Mode Matching in Gain-assisted Double-plasmonic Resonance Nanostructure

**DOI:** 10.1038/s41598-017-10243-y

**Published:** 2017-08-29

**Authors:** Gui-Ming Pan, Da-Jie Yang, Li Zhou, Zhong-Hua Hao, Qu-Quan Wang

**Affiliations:** 1 Key Laboratory of Artificial Micro- and Nano-structures of the Ministry of Education, School of Physics and Technology, Wuhan University, Wuhan, 430072 P.R. China; 2 The Institute for Advanced Studies, Wuhan University, Wuhan, 430072 P.R. China

## Abstract

We theoretically study the gain-assisted double plasmonic resonances to enhance second harmonic generation (SHG) in a centrosymmetric multilayered silver-dielectric-gold-dielectric (SDGD) nanostructure. Introducing gain media into the dielectric layers can not only compensate the dissipation and lead to giant amplification of surface plasmons (SPs), but also excite local quadrupolar plasmon which can boost SHG by mode matching. Specifically, as the quadrupolar mode dominates SHG in our nanostructure, under the mode matching condition, the intensity of second harmonic near-field can be enhanced by 4.43 × 10^2^ and 1.21 × 10^5^ times when the super-resonance is matched only at the second harmonic (SH) frequency or fundamental frequency, respectively. Moreover, the intensity of SHG near-field is enhanced by as high as 6.55 × 10^7^ times when the nanostructure is tuned to double super-resonances at both fundamental and SH frequencies. The findings in this work have potential applications in the design of nanosensors and nanolasers.

## Introduction

Second harmonic generation has been extensively studied both theoretically and experimentally in the past decades due to the wide applications^1–7^. Especially, finding an efficient method to enhance SHG has attracted much attention^8–11^. In recent years, plasmonic structures coupled with nonlinear materials draw intense attention in SHG enhancement^12–18^. Surface plasmon (SP) resonance of metal structures can greatly concentrate the local field and lead to significantly enhanced linear and nonlinear optical processes. Since the intensity of second harmonic (SH) field is directly related to the square of the fundamental field intensity, it can be greatly enhanced by achieving the SP resonance at the fundamental frequency. Pu *et al*. have proposed a coupled system of the nonlinear material with the plasmonic metal structure to highlight the SHG signal by enhancing the near field around nonlinear material^12^. However, the efficiency of SHG seriously suffers from the radiative losses of plasmons^19^. Employing Fano or magnetic plasmon resonances to avoid scattering and nonradiative losses at fundamental frequency has been proved as an efficient way to enhance SHG^20–23^. Moreover, when two plasmonic resonance modes are properly matched to both the fundamental and SH frequencies, the SH emission can be further enhanced^16, 24, 25^.

On another front, introducing active materials (such as dye molecules, rare earth ions, or semiconductor nanocrystals) is an effective method to reduce or compensate the intrinsic losses in plasmonic structures^26–31^. In an active nanostructure, when the gain coefficient approaches a critical value, which is called gain threshold, the plasmonic losses can be exactly compensated and thus super-resonance occurs^28, 32, 33^. At the gain threshold, the strong coupling between the gain medium and the metal structure will transfer the energy from the gain medium to the SP modes at the resonant frequencies. The quality factor of the plasmonic system is significantly enhanced. The scattering and absorption optical cross sections and the electric near-field will be enhanced dramatically. Surface plasmon super-resonance in single mode is well studied. And Wu *et al*. have designed a three-layered bimetallic Ag-Au nanoshell, which can support two similar super-resonance SP modes^34, 35^.

Here, we design a spherical SDGD multi-layered nanostructure and investigate the plasmon-enhanced SHG through gain-assisted double-plasmonic resonances. We show that the dark quadrupolar mode can be excited by introducing gain media into the SDGD nanostructure. Meanwhile, two tunable super-resonances of high-energy quadrupolar mode (dark mode) and low-energy dipolar mode (bright mode) can be excited simultaneously as the gain coefficients are adjusted to the appropriate values. The super-resonance frequencies can be tuned in a wide range via altering the structural parameters. In centrosymmetric nanostructures, the SH emission is generated by retardation effect of the local plasmon at fundamental frequency^36^, and can be greatly enhanced when the high-energy quadrupolar mode and low-energy dipolar mode are adjusted to match the SH and fundamental frequencies simultaneously. The mode-matched double-resonant plasmonic nanoshells can serve as an ideal candidate for SHG enhancement.

## Numerical Results and Discussion

In this paper, we employ Mie scattering theory in multilayered spheres^37–40^ to calculate the mode decomposition. The optical extinction and scattering factors are expressed as,

1 $${Q}_{ext}=\frac{2}{kr}\sum _{n=1}^{\infty }(2n+1){\rm{Re}}\{{a}_{n}+{b}_{n}\},$$

2 $${Q}_{sca}=\frac{2}{kr}\sum _{n=1}^{\infty }(2n+1)({|{a}_{n}|}^{2}+{|{b}_{n}|}^{2}),$$

where, *k* is the propagation constant, *r* is the outer radius of the particle, *a*_*n*_ and *b*_*n*_ are the scattering coefficients. Besides, *a*_*n*_ and *b*_*n*_ dominate electric and magnetic components, respectively. *a*_1_, *a*_2_, … are electric dipole, electric quadruple, … and *b*_1_, *b*_2_, … are magnetic dipole, magnetic quadruple, … The extinction and scattering cross sections are given as *C*_*ext*_ = *Q*_*ext*_ · *πr* ^2^ and *C*_*sca*_ = *Q*_*sca*_ · *πr* ^2^.

SHG problems are solved by using the finite element method (FEM) in a commercial software (COMSOL Multiphysics), which is complex in Mie scattering theory for the four-layered nanoshells. The optical cross sections in Comsol calculation are defined as ref. 41,

3 $${C}_{sca}=\frac{1}{{I}_{0}}\iint \vec{n}\cdot {\vec{s}}_{sca}ds,$$

4 $${C}_{abs}=\frac{1}{{I}_{0}}\iiint QdV,$$

where $\vec{n}$ is the normal vector pointing outwards from the nanodot, ${\vec{S}}_{sca}$ is the Poynting Vector, *Q* is the power loss density, and *I*_0_ is the intensity of incident beam. The scattering cross section is the integral taken over the closed surface of the nanoparticle, and the absorption cross section is the integral taken over the volume of the nanoparticle. The extinction cross section is simply the sum of the scattering and absorption cross sections *C*_ext_ = *C*_sca_ + *C*_abs_. The optical extinction cross section calculated by FEM is perfectly fit with that calculated by Mie scattering theory (Figures S1, Supporting Information).

The second-order nonlinear process is forbidden in the dipolar approximation in centrosymmetric materials^5^, such as gold and silver. The nonlinear response is dominated by the contribution of the broken centrosymmetry of metal surface and the field gradients^42^. The nonlinear polarization can be replaced by external current density. The external current density component perpendicular to the particle surface can be expressed as refs 43 and 44,

5 $${J}_{{\rm{surf}},\perp }(\vec{{\rm{r}}},2\omega )=\partial {P}_{{\rm{surf}},\perp }(\vec{{\rm{r}}},2\omega )/\partial t,$$

where ${P}_{{\rm{surf}},\perp }(\overrightarrow{r},2\omega )$ is the second order polarization perpendicular to the particle surface and is defined as ref. 44,

6 $${P}_{{\rm{surf}},\perp }(\vec{{\rm{r}}},2\omega )={\chi }_{\mathrm{surf},\perp \perp \perp }{E}_{\perp }^{2}(\vec{{\rm{r}}},\omega ).$$

In Eq. (6), *E*_⊥_ is the perpendicular component fundamental electric filed, and ${\chi }_{\mathrm{surf},\perp \perp \perp }$ ${\chi }_{\mathrm{surf},\perp \perp \perp }=$ $-\,a[{\varepsilon }_{r}(\omega )-1]e{\varepsilon }_{0}/4m{\omega }^{2}$is the perpendicular component of the second order susceptibility (*a* = 1)^45^. Then, Eq. (5) can be rewritten in the equivalent computer-convenient form,

7 $${J}_{{\rm{surf}},\perp }(\vec{{\rm{r}}},2\omega )=i2\omega {\varepsilon }_{0}{\chi }_{\mathrm{surf},\perp \perp \perp }{E}_{\perp }^{2}(\vec{{\rm{r}}},\omega ),$$

where, $i=\sqrt{-1}$. The SHG field can be obtained by solving the Maxwell equations in the weakened form.

**Figure 1:**
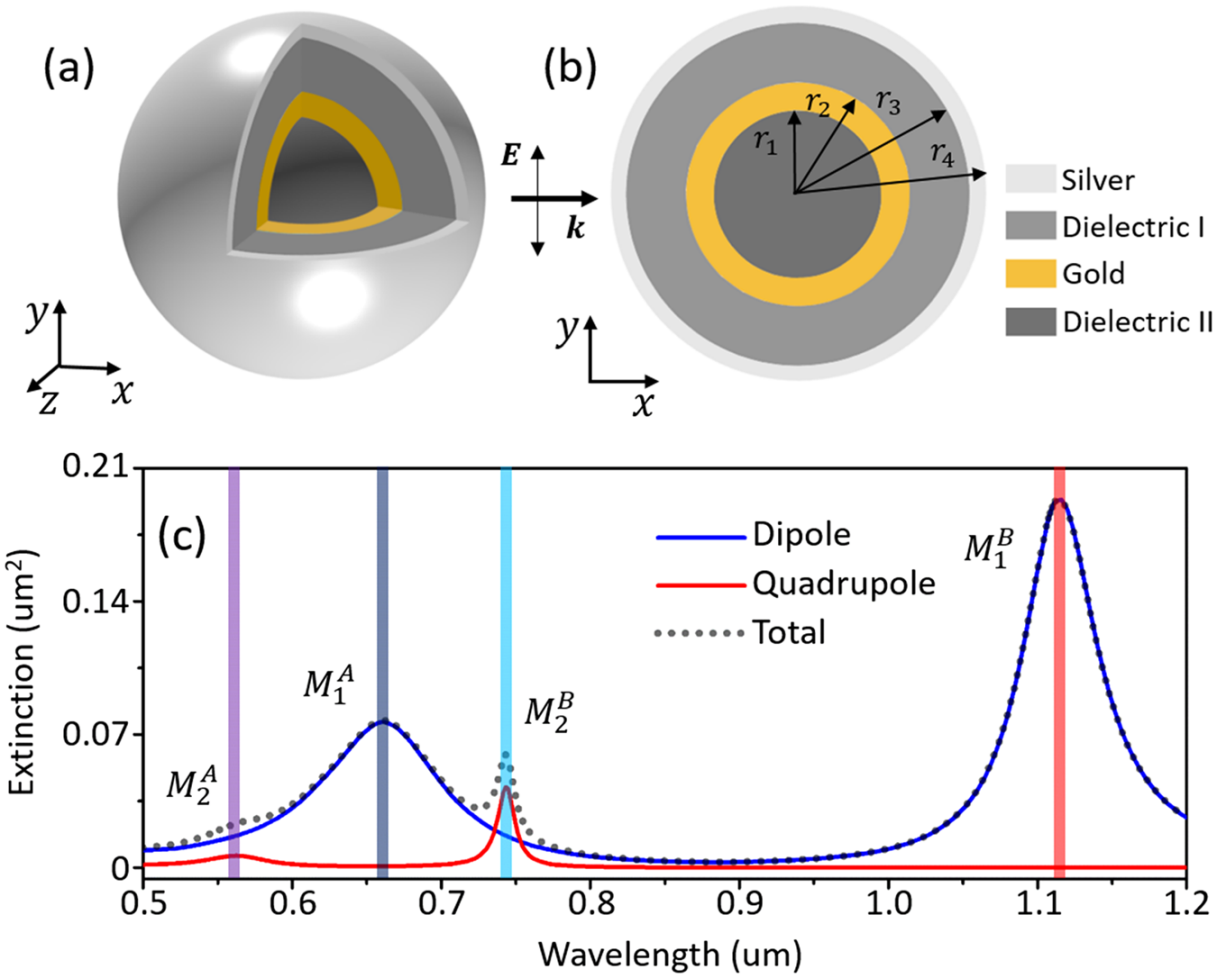
(**a**) Schematic diagram and (**b**) sectional view of the SDGD nanostructure. (**c**) Extinction cross section spectrum and mode decomposition of the passive SDGD nanostructure. The blue curve is dipole extinction, the red curve is quadrupole extinction, and the gray short dot line is the total extinction cross section.

The multilayered SDGD structure is illustrated in Fig. 1a and b. The outermost layer is silver. And the gold nanoshell is surrounded by two dielectrics, where the gain medium can be introduced by doping. The incident fundamental wave is along *x*-axis (*k*) and the polarization direction is along *y*-axis (*E*). The amplitude of the fundamental electric field is set as 1.0 V/m. The refractive index of the surrounding medium is taken to be 1.43. The real parts of the refractive index are 1.43 for Dielectric I and 1.69 for Dielectric II, respectively^46, 47^. The imaginary parts of the two dielectric regions are determined by the dopant of some rare-earth ions or dyes^48^, such as Pr^3+^, Ho^3+^, Er^3+^, Nd^3+^, Tm^3+^ and Oregon Green 488 (OG 488)^49, 50^. The gain coefficient of doped Dielectric I is marked as *κ*_1_ and the gain coefficient of Dielectric II is marked as *κ*_2_. In general, the gain coefficients can be tuned by modifying the doping concentration. We assume that the gain emission exists in all the optical frequencies (the results with the gain media having characteristic emission frequencies are shown in Figures S5 and S6, Supporting Information). The refractive index data describing the optical responses of silver and gold are given by Johnson and Christy^51^.

First, we consider a passive SDGD nanostructure (without gain media). The radii of different nanoshells are taken as *r*_1_ = 27 nm, *r*_2_ = 36 nm, *r*_3_ = 55.2 nm and *r*_4_ = 60.2 nm, respectively. The maximum element size of FEM mesh is 6 nm which is sufficiently accurate (Figures S1, Supporting Information). The spectrum of extinction cross section and mode decomposition of the SDGD system are shown in Fig. 1(c). The dipolar modes are marked as ${M}_{1}^{A}$ and ${M}_{1}^{B}$, respectively. The quadrupolar modes are marked as ${M}_{2}^{A}$ and ${M}_{2}^{B}$, respectively. However, in the passive nanostructure, the quadrupolar part in ${M}_{2}^{A}$ mode is very weak and almost merged into the dipole background.

**Figure 2:**
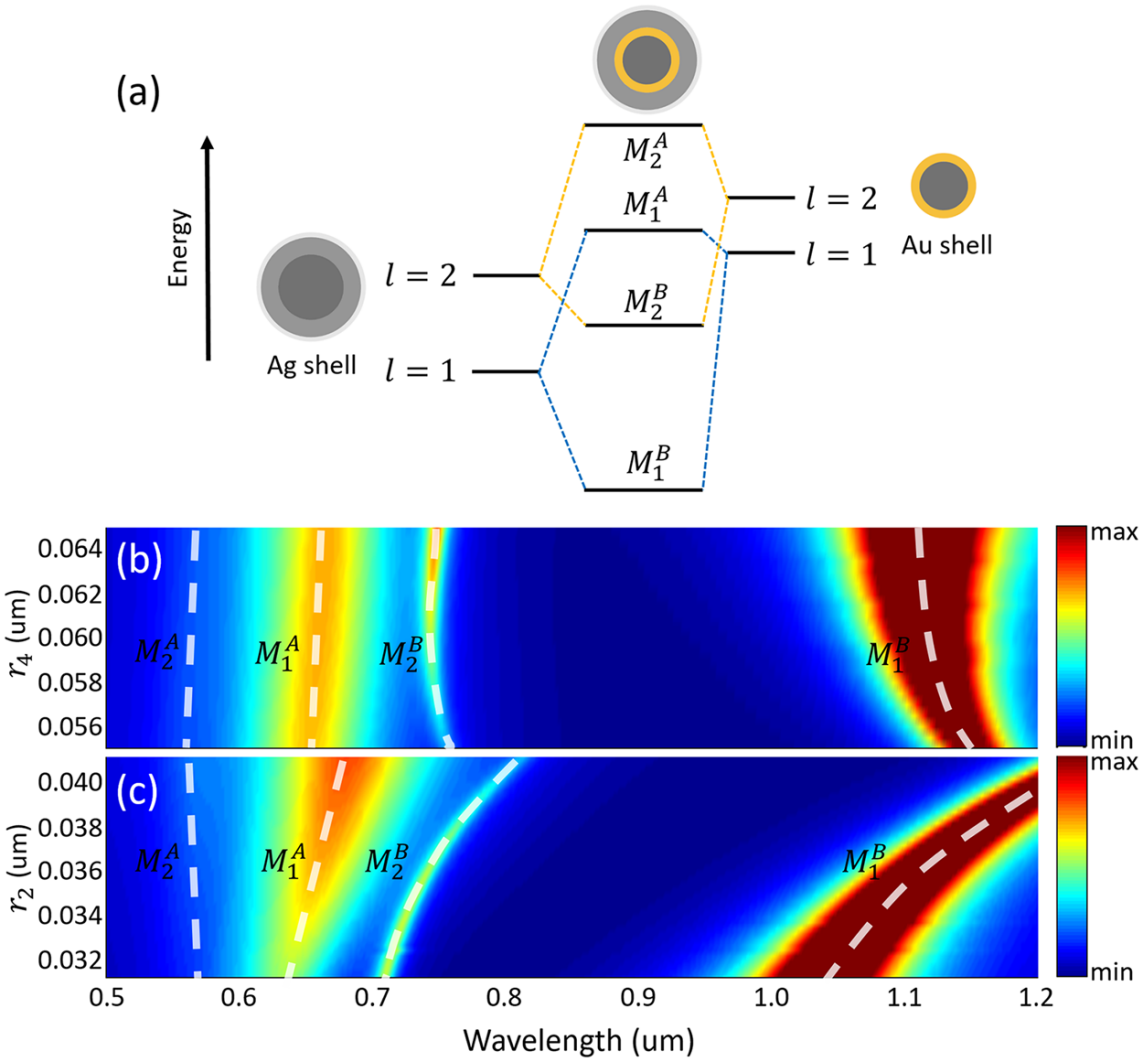
(**a**) Illustration of the energy level diagram and modes hybridization of silver and gold nanoshells in the SDGD nanostructure. Contour plot of the extinction cross sections as a function of wavelength and radius of the silver nanoshell (**b**) and gold nanoshell (**c**).

The optical responses of the SDGD nanostructure with four SP resonance modes are revealed through the theory of plasmon hybridization. As shown in Fig. 2a, *l* denotes angular momenta. Specifically, *l* = 1 denotes dipolar mode and *l* = 2 denotes quadrupolar mode. In spherical symmetric nanostructure, the hybridization between different angular momenta modes is forbidden^52–55^. The dipolar mode (*l* = 1) of the silver nanoshell can only interact with the dipolar mode (*l* = 1) of the gold nanoshell. Similarly, the quadrupolar mode of the silver nanoshell can only interact with the quadrupolar mode of the gold nanoshell. The mode hybridization makes the antibonding modes blue shift and the bonding modes red shift. The ${M}_{1}^{A}$ and ${M}_{1}^{B}$ (${M}_{2}^{A}$ and ${M}_{2}^{B}$) modes are corresponding to the bonding and antibonding dipolar (quadrupolar) modes, respectively. The frequencies of the SP resonant modes can be tuned in a wide range by changing the size parameters of the SDGD nanostructure. As shown in Fig. 2b, we assume that the thicknesses of the silver and gold nanoshells are invariable. When keeping the radius of gold nanoshell (*r*_2_) unchanged and increasing the radius of the silver nanoshell (*r*_4_), the antibonding modes (${M}_{2}^{A}$ and ${M}_{1}^{A}$) will be red shifted, and the bonding modes (${M}_{2}^{B}$ and ${M}_{1}^{B}$ modes) will be blue shifted, because the interaction between silver and gold nanoshells becomes weaken. As shown in Fig. 2c, if keeping the radius of silver nanoshell unchanged and increasing the radius of gold nanoshell, the bonding modes are red shifted as the strength of mode hybridization is increased. However, the antibonding modes are also slightly red shifted because the eigenstates of the gold nanoshell are red shifted.

**Figure 3:**
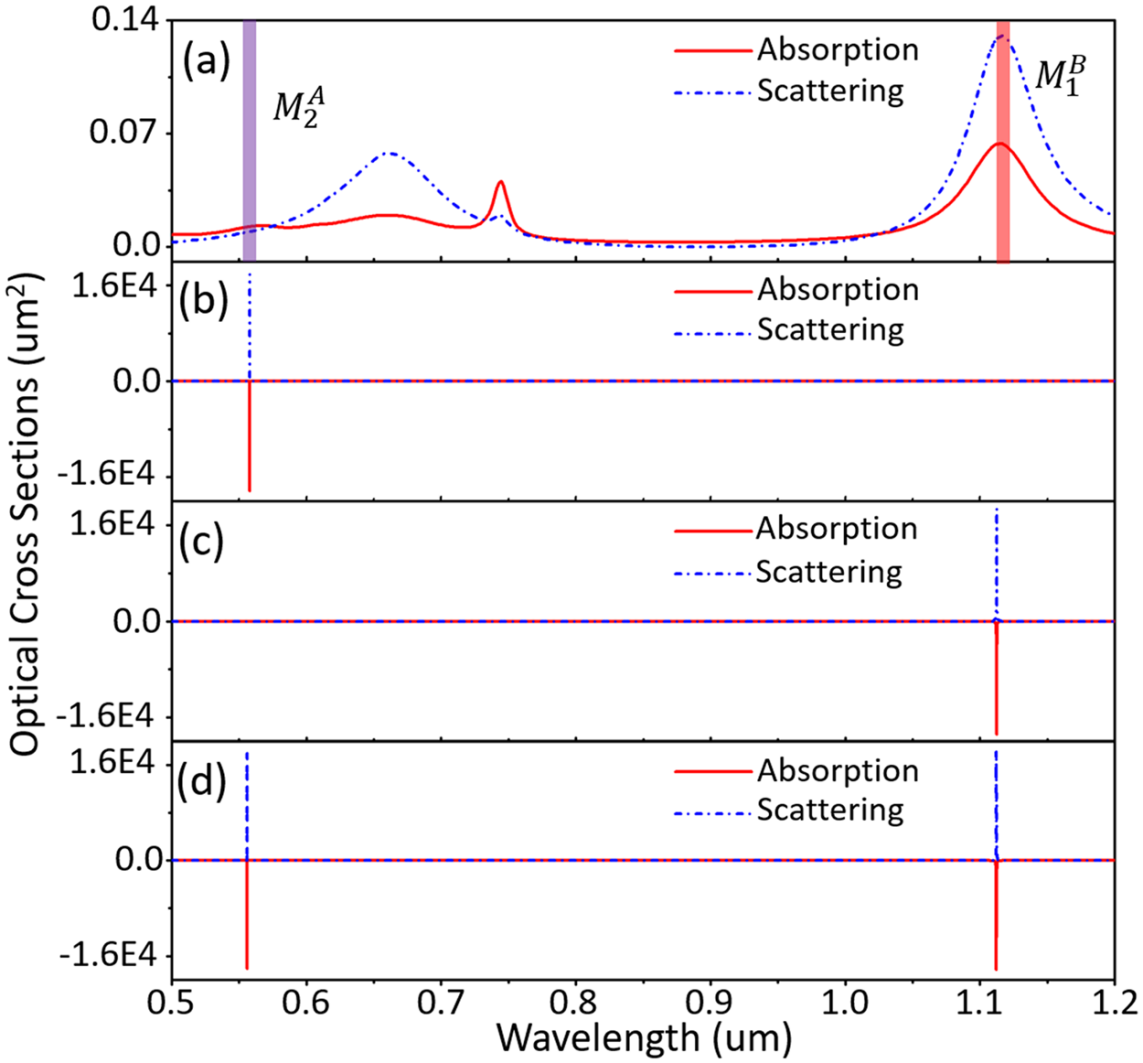
(**a**) Absorption and scattering cross sections of the passive SDGD nanostructure. Super-resonances at ${M}_{2}^{A}$ mode (*κ*_1_ = 0.145 and *κ*_2_ = 0.400) (**b**), ${M}_{1}^{B}$ mode (*κ*_1_ = 0.070 and *κ*_2_ = 0.400) (**c**), both of ${M}_{2}^{A}$ and ${M}_{1}^{B}$ modes (*κ*_1_ = 0.068 and *κ*_2_ = 0.510) (**d**) in the gain-assisted SDGD nanostructure.

When gain is introduced into the Dielectric I and Dielectric II, the losses of ${M}_{2}^{A}$ and ${M}_{1}^{B}$ modes can be exactly compensated by tuning the values of the gain coefficients *κ*_1_ and *κ*_2_, leading to super-resonance at either one or both two modes (Fig. 3). The gain coefficients used in this work are in the reasonable range of gain medium, which can be adjusted by doping concentration^48, 56, 57^. According to Fig. 3, the shift of resonant frequency induced by different gain coefficients is very small and can be neglected, which suggests that the gain almost has no implications for the plasmon hybridization. As mentioned before, these results are under the assumption that the gain emission spectrum of the gain media cover both of ${M}_{1}^{B}$ and ${M}_{2}^{A}$ modes. The gain thresholds of *κ*_1_ and *κ*_2_ for ${M}_{2}^{A}$ and ${M}_{1}^{B}$ modes are shown in Figure S3 in Supporting Information.

**Figure 4:**
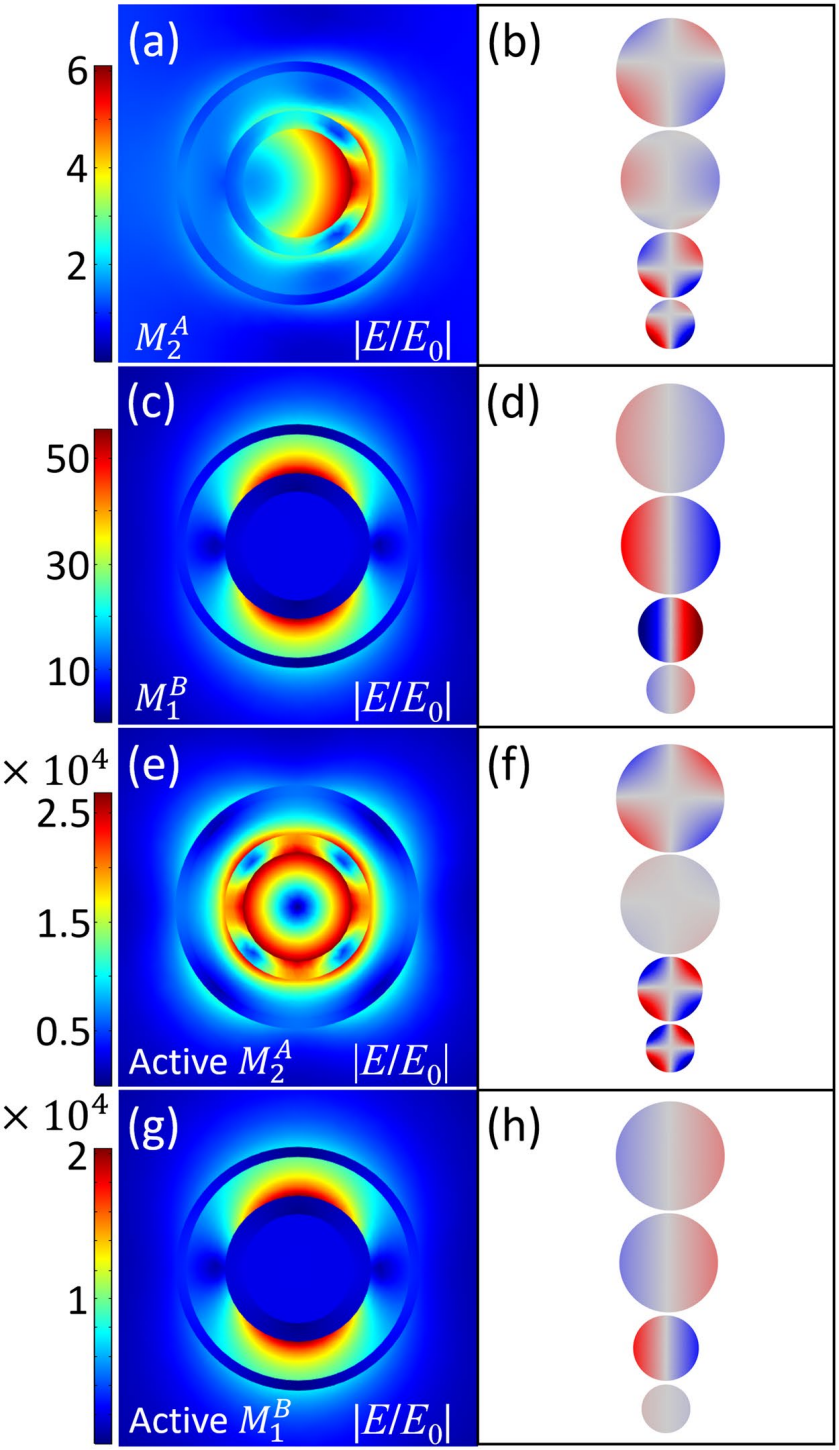
Local electric near-field plotted in x-y plane (left column) and corresponding surface charge distributions (right column) of ${M}_{2}^{A}$ and ${M}_{1}^{B}$ modes. (**a**) and (**c**) are near-field distributions of ${M}_{2}^{A}$ and ${M}_{1}^{B}$ modes in passive nanostructure. (**e**) and (**g**) are near-field distributions of ${M}_{2}^{A}$ and ${M}_{1}^{B}$ modes in active nanostructure. The right column (**b**,**d**,**f**,**h**) are corresponding surface charge distributions of the near-field in left column (**a**,**c**,**e**,**g**) respectively. The four spheres (from up to down) in each right column figures denote the charge distributions at the external, internal surface of silver shell and the external, internal surface of gold shell, respectively.

The corresponding near-field distributions of ${M}_{2}^{A}$ and ${M}_{1}^{B}$ modes in the passive and active nanostructures are shown in Fig. 4. In the active nanostructure, the near-fields of ${M}_{2}^{A}$ and ${M}_{1}^{B}$ modes are both extremely enhanced when the two SP modes reach to their super-resonances. The near-field intensity can be enhanced by nearly 2.69 × 10^4^ times at ${M}_{2}^{A}$ mode (Fig. 4e) and 2.04 × 10^4^ times at ${M}_{1}^{B}$ mode (Fig. 4g). In the passive nanostructures, the retardation effect at ${M}_{2}^{A}$ mode is clearly observed (Fig. 4a). Especially, the great retardation effect at ${M}_{2}^{A}$ leads to quadrupolar polarization of the local plasmon. For ${M}_{1}^{B}$ mode (Fig. 4c and g), the retardation effect is not obvious. According to Fig. 1c, as ${M}_{2}^{A}$ mode is an intrinsically quadrupolar mode. When gain is introduced into the nanostructure, the quadrupole ${M}_{2}^{A}$ mode is greatly enhanced, leading to a symmetric near-field distribution in Fig. 4e. The near-field distribution at ${M}_{2}^{A}$ mode is greatly influenced by gain (Figures S4, Supporting Information).

To further study the electromagnetic features of the nanostructure, we also calculate the surface charge distributions, which is shown in the right column in Fig. 4. The surface charge distributions can be calculated by the boundary conditions of electromagnetic field^58^,

8 $$\vec{n}\cdot ({\vec{E}}_{I}-{\vec{E}}_{II})=\frac{\rho }{{\varepsilon }_{sur}},$$

where $\vec{n}$ is the normal vector, ${\vec{E}}_{I}$ and ${\vec{E}}_{II}$ are electric field of the contact surfaces, *ρ* is the surface electric charge density, and *ε*_*sur*_ is the dielectric constant. The charge distribution shown in Fig. 4b suggests that in the passive nanostructure, the quadrupolar polarization derives from retardation effect. The gain media can tune the resonant phase. According to Fig. 4f, there is no phase retardation when ${M}_{2}^{A}$ comes to super-resonance, which can be defined as “pure quadrupolar polarization” (PQP). Besides, Fig. 4f indicates that ${M}_{2}^{A}$ (at gain threshold) cannot couple with the polarized light directly and is referred to dark mode. As the modes with the same angular momenta can couple with each other, PQP leads to perfect mode matching with the quadrupole SH emission. The gain media also modify the resonant phase of ${M}_{1}^{B}$ mode. Comparing with the passive ${M}_{1}^{B}$ in Fig. 4d, the resonant phase is reversed in active ${M}_{1}^{B}$ shown in Fig. 4h. Both passive and active ${M}_{1}^{B}$ modes are dipolar polarization.

The SH emission in the spherical particles has been studied excessively^59–61^. Retardation effects is crucial for SHG from the centrosymmetric nanostructures^62^. According to the selection rules, the SH emission channel *E*_1_ + *E*_1_ → *E*_1_ is forbidden in centrosymmetric nanostructures^5, 59^. In consideration of our nanostructure, the SH emission can be only through the channels of *E*_1_ + *E*_1_ → *E*_2_ and *E*_1_ + *E*_1_ → *E*_3_, where *E*_1_, *E*_2_ and *E*_3_ correspond to the electric dipolar, quadrupolar and octupolar excitations, respectively. The ratio of ${P}_{2}^{2\omega }/{P}^{2\omega }$ is always much larger than ${P}_{3}^{2\omega }/{P}^{2\omega }$, where *P* ^2*ω*^ is the total SH power, ${P}_{2}^{2\omega }$ is the quadrupole term, and ${P}_{3}^{2\omega }$ is the octupolar term^63^. According to the surface charge distributions in Fig. 4f, ${M}_{2}^{A}$ mode shows quadrupolar polarization in active nanostructure, which means that the gain media not only enhance the near-fields at both of ${M}_{2}^{A}$ and ${M}_{1}^{B}$ modes, but also help to excite PQP. And ${M}_{2}^{A}$ mode at 2*ω* is used to boost the SH emission process as it matches with the quadrupolar mode of SH emission.

**Figure 5:**
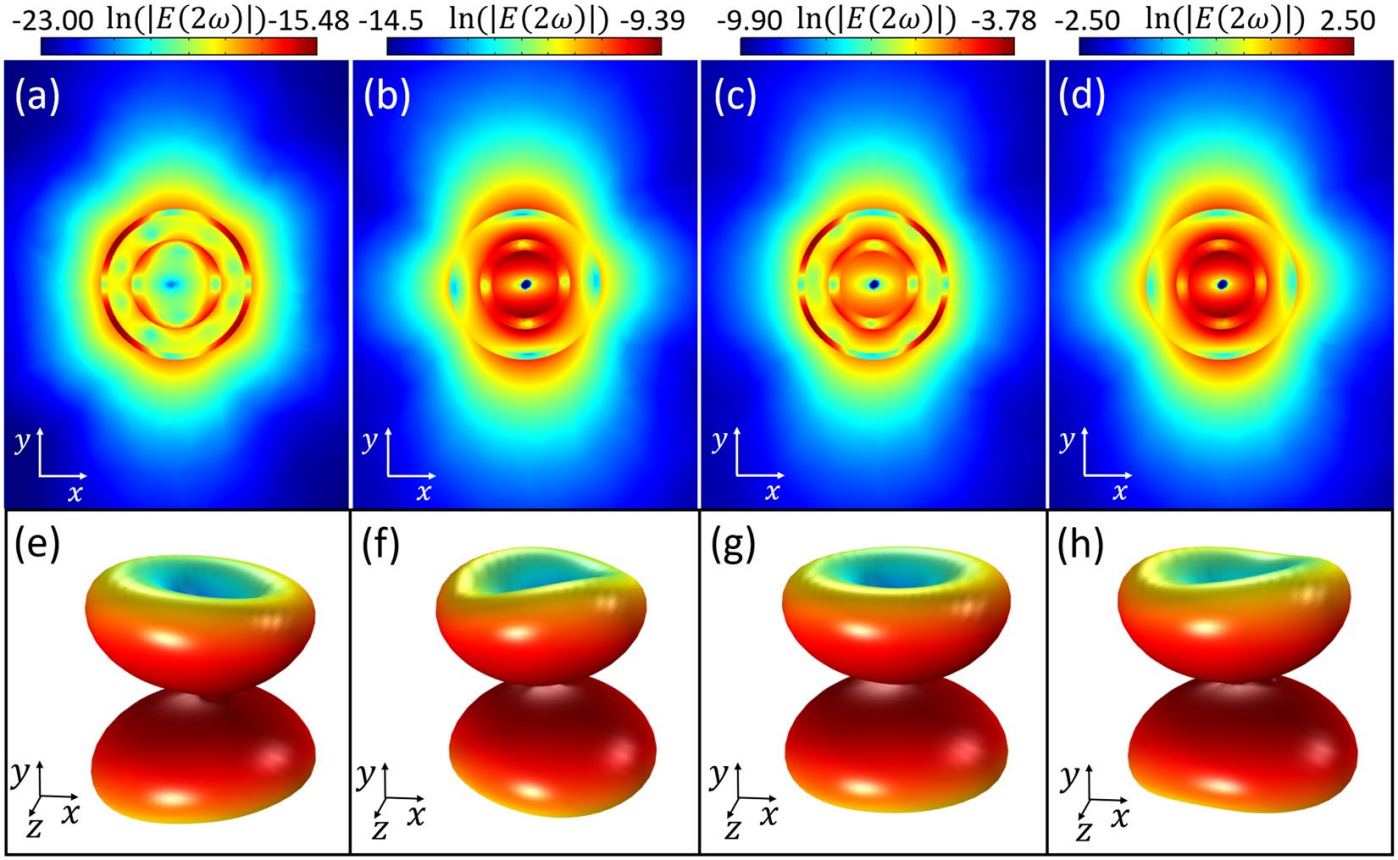
Near-field distributions of the SH intensity plotted on logarithmic scale (ln(|*E*(2ω)|)) and the SH emission patterns. (**a**) Passive nanostructure, (**b**) Active nanostructure with super-resonance only at ${M}_{2}^{A}$ mode, (**c**) Active nanostructure with super-resonance only at ${M}_{1}^{B}$ mode, (**d**) Active nanostructure with super-resonances at both of ${M}_{2}^{A}$ and ${M}_{1}^{B}$ modes. (**e**–**h**) The corresponding 3D far-field distributions in (**a**–**d**). All of the SH emission are quadrupole.

The harmonic field distributions in logarithmic scale are shown in Fig. 5. Due to the retardation effect, one can see octupole terms of SHG according to the field distributions shown in Fig. 5a and c. However, the octupole terms nearly do not exist in the cases of Fig. 5b and d. The quadrupole terms of SHG can be enhanced extremely due to the strong coupling between the quadrupole term of SHG and the local plasmon ${M}_{2}^{A}$ mode (quadrupolar polarization). The proportion of the octupole term drops to a very small level and can be neglected. Specifically, comparing with the passive nanostructure, the intensity of SH near-field is enhanced by 4.43 × 10^2^ times when the SH emission couples to the gain-assisted local plasmonic super-resonance only at SH frequency. The intensity of SH near-field is enhanced by 1.21 × 10^5^ times when the gain-assisted local plasmonic super-resonance only occurs at fundamental frequency (Fig. 5c). The intensity of SH near-field is enhanced by 6.55 × 10^7^ times when the super-resonances at SH and fundamental frequency are both excited by gain media (Fig. 5d). The far-field patterns (Fig. 5e–h) demonstrate that the SH emission is quadrupolar polarization.

**Figure 6:**
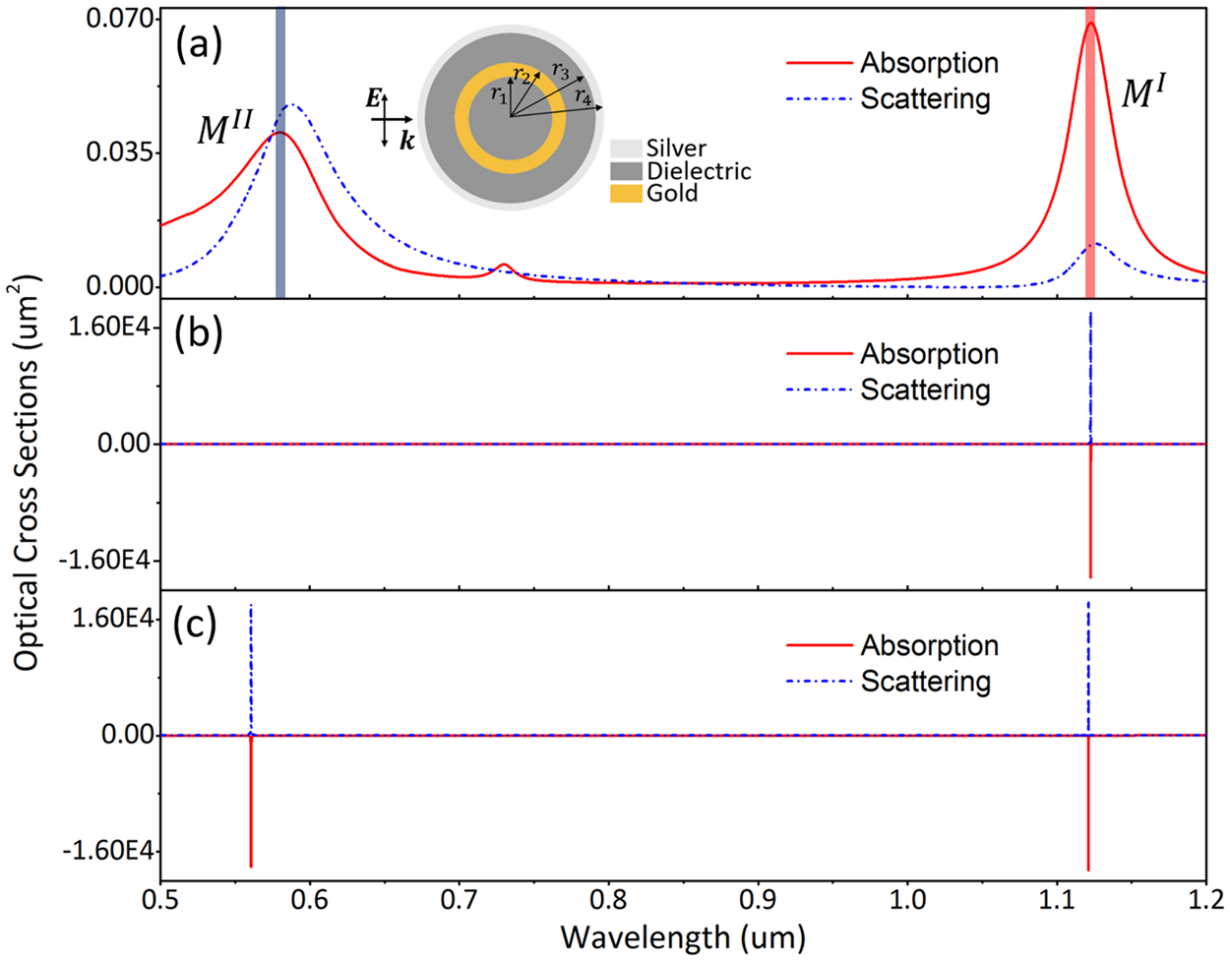
(**a**) Absorption and scattering cross sections of the passive SDGD nanostructure. Super-resonances at *M* ^*I*^ mode (*κ*_I_ = 0.0174 and *κ*_II_ = 0.600) (**b**), both of *M* ^*I*^ and *M* ^*II*^ modes (*κ*_I_ = 0.0125 and *κ*_II_ = 0.858) (**c**) in the gain-assisted nanostructure.

**Figure 7:**
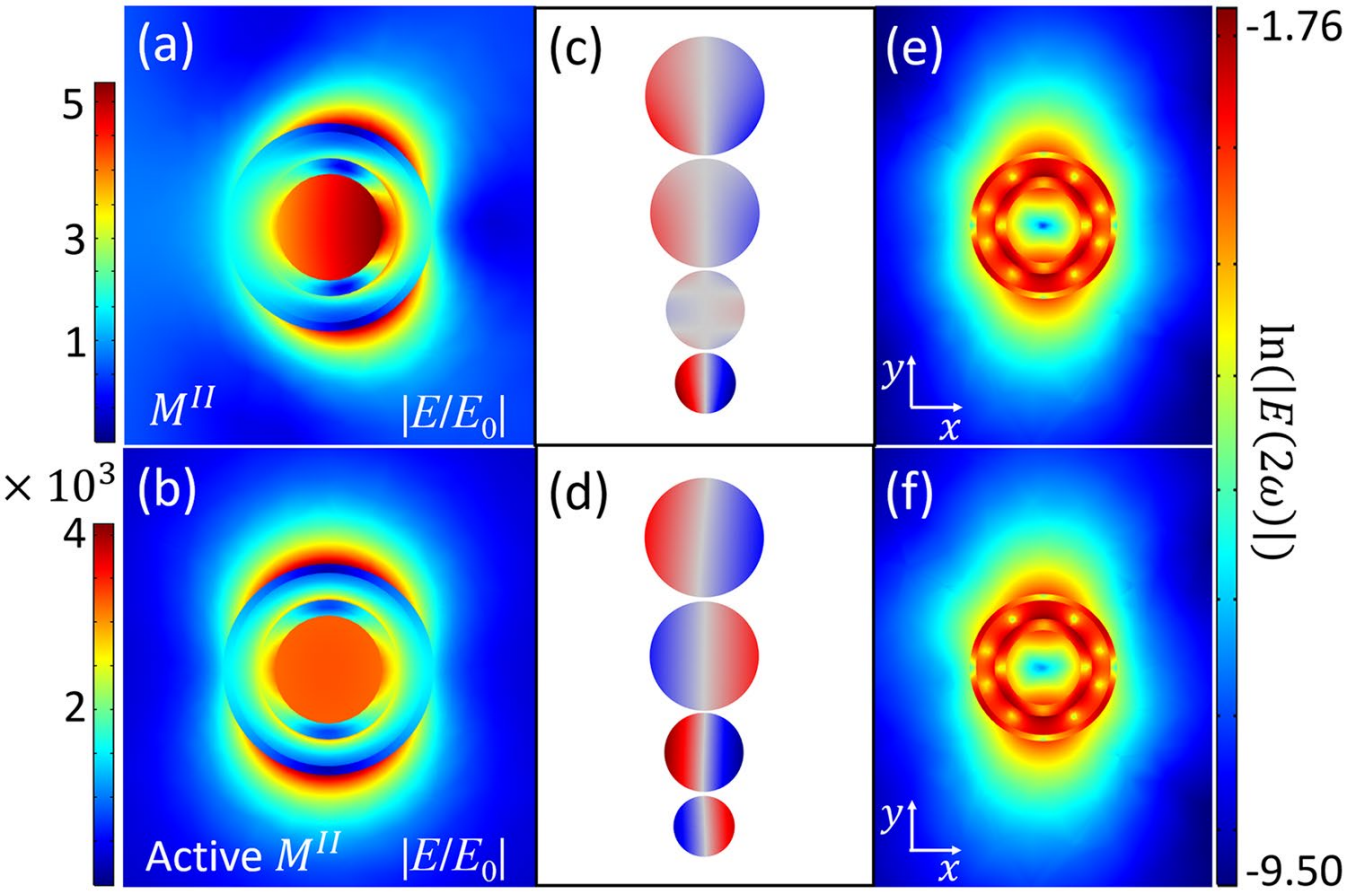
Near-field distributions of passive and active *M* ^*II*^ modes plotted in x-y plane (**a**,**b**). Surface charge distributions of passive and active *M* ^*II*^ modes (**c**,**d**). Near-field distributions of the SH intensity plotted on logarithmic scale (ln(|*E*(2ω)|)) (**e**,**f**). (**e**) is super-resonance of local plasmon at only fundamental frequency and (**f**) is super-resonance of local plasmon at fundamental and SH frequency.

In order to highlight the mode matching in SH frequency, the super-resonance of dipolar mode at SH frequency for SHG enhancement is shown in Figs 6 and 7. A case of SDGD nanostructure with *r*_1_ = 30.7 nm, *r*_2_ = 39.7 nm, *r*_3_ = 55 nm and *r*_4_ = 60 nm is designed. The surrounding media is air with refractive index of 1. The two dielectric regions have the refractive index of 1.43. The gain coefficients of the outer and inner dielectric layers are marked as *κ*_I_ and *κ*_II_, respectively. The modified nanostructure owns two plasmonic modes marked as *M* ^*I*^ and *M* ^*II*^, which are tuned to the fundamental and SH frequencies and can be excited respectively or simultaneously.

As shown in Fig. 7a and b, the near-field can be extremely enhanced in the active *M* ^*II*^ mode. However, unlike the ${M}_{2}^{A}$ mode, the surface charge distributions in Fig. 7c and d suggest that the *M* ^*II*^ mode is a dipolar polarization in both of the passive and the active nanostructures. The local plasmon with dipolar polarization cannot couple with the SHG (mainly quadrupolar emission). As shown in Fig. 7e and f, the SHG emission in the active nanostructure is not enhanced, which shows the local plasmon with dipolar polarization at SH frequency has no contribution to the SHG enhancement.

## Conclusion

We have designed a high-efficiency nanostructure to enhance the SHG and revealed a significant approach to take advantage of the mode-matched plasmon at SH frequency for the SHG enhancement in the centrosymmetric nanostructures. The resonant modes in the SDGD nanostructure are tunable and the gain media induce the double super-resonances simultaneously. Besides, the ‘PQP’ can be excited in the ${M}_{2}^{A}$ mode, which satisfies the mode matching as the SH emission is quadrupole in our system. Specifically, the second harmonic field is enhanced by 1.21 × 10^5^ times with the super-resonance at the fundamental frequency, 4.43 × 10^2^ times with the super-resonance at the SH frequency, and 6.55 × 10^7^ times with the double super-resonances at both of the fundamental and SH frequencies. The presented active nanostructures provide a clear insight into the contribution of SHG enhancement at both the fundamental and the SH frequencies.

## Electronic supplementary material

Supplementary Information
