# Supplementary material for: Enhanced Second Harmonic Generation by Mode Matching in Gain-assisted Double-plasmonic Resonance Nanostructure
Source: Sci Rep. 2017 Aug 29;7:9776. doi: 10.1038/s41598-017-10243-y (PMC5575406; doi:10.1038/s41598-017-10243-y)
Supplement: Supplementary file 1 — Supplementary Information [file 41598_2017_10243_MOESM1_ESM.doc]

Supplementary Information

**Enhanced Second Harmonic Generation by Mode Matching in Gain-assisted Double-plasmonic Resonance Nanostructure**

Gui**-**Ming Pan^1^, Da-Jie Yang^1,2^, Li Zhou^1,*^, Zhong**-**Hua Hao^1,*^, Qu**-**Quan Wang^1,2,*^

^1^Key Laboratory of Artificial Micro- and Nano-structures of the Ministry of Education, School of Physics and Technology, Wuhan University, Wuhan 430072, P. R. China.
^2^The Institute for Advanced Studies, Wuhan University, Wuhan 430072, P. R. China.

1. **Comparing the calculation results of FEM and Mie scattering theory.**

**
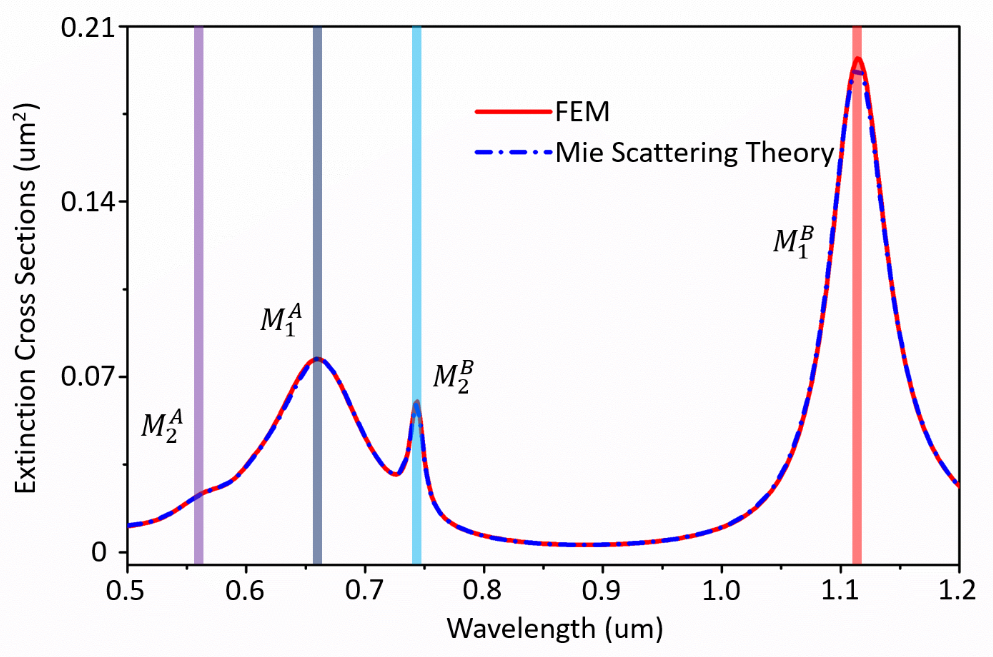
**

**Figure S1.** Extinction cross sections calculated by FEM and Mie scattering theory. FEM calculation result is in agreement with Mie scattering theory in this work.

1. **Modes decomposition.**


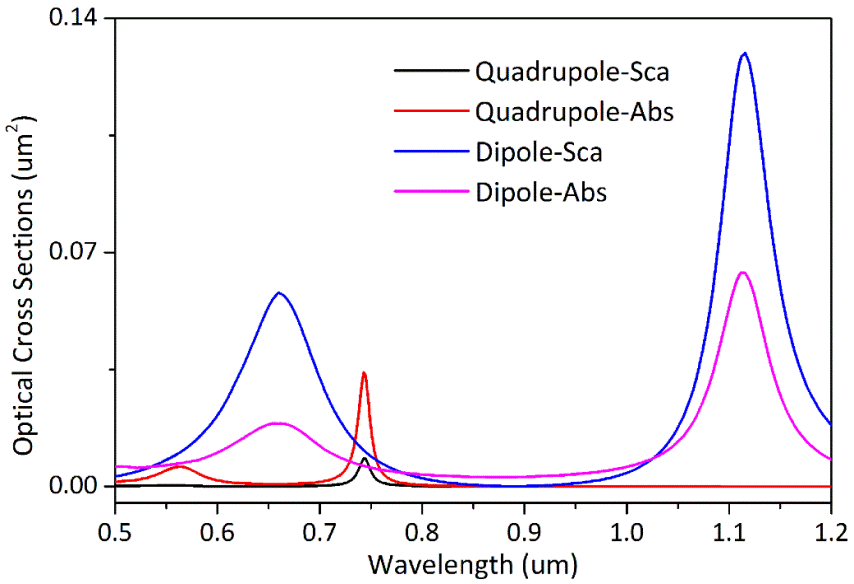


**Figure S2.** Optical cross section spectra and mode decomposition of the passive SDGD nanostructure.

Mie scattering theory is a very powerful method to solve electromagnetic problem in spherical particles. According to the equations (1) and (2) in the main article, the extinction and scattering factors of dipolar and quadrupolar components can be expressed as

$Q_{ext-dipole}=\frac{6}{kr}\mathrm{Re}\left\{a_{1}+b_{1}\right\}$, $Q_{sca-dipole}=\frac{6}{kr}(\left|a_{1}\right|^{2}+\left|b_{1}\right|^{2})$, (S1)

$Q_{ext-quad}=\frac{10}{kr}\mathrm{Re}\left\{a_{2}+b_{2}\right\}$, $Q_{sca-quad}=\frac{10}{kr}(\left|a_{2}\right|^{2}+\left|b_{2}\right|^{2})$, (S2)

where, *Q_ext-dipole_* and *Q_ext-quad_* are dipolar and quadrupolar extinction factors respectively; *r* = *r*_4_. The extinction cross sections $C_{ext}=Q_{ext}\cdot\pi r^{2}$, $C_{sca}=Q_{sca}\cdot\pi r^{2}$, and $C_{abs}=C_{ext}-C_{sca}$. In multilayered sphere, the scattering coefficients can be obtained as

$a_{n}=a_{n}^{L+1}=\frac{[H_{n}^{a}(m_{L}x_{L})/m_{L}+n/x_{L}]\psi_{n}(x_{L})-\psi_{n-1}(x_{L})}{[H_{n}^{a}(m_{L}x_{L})/m_{L}+n/x_{L}]\xi_{n}(x_{L})-\xi_{n-1}(x_{L})}$, (S3)

$b_{n}=b_{n}^{L+1}=\frac{[m_{L}H_{n}^{b}(m_{L}x_{L})+n/x_{L}]\psi_{n}(x_{L})-\psi_{n-1}(x_{L})}{[m_{L}H_{n}^{b}(m_{L}x_{L})+n/x_{L}]\xi_{n}(x_{L})-\xi_{n-1}(x_{L})}$, (*n* = 1, 2 and *L* = 4), (S4)

where, $\psi_{n}$ and $\xi_{n}$ are the Riccati-Bessel functions; $x_{L}\equiv kr_{L}$; and *m_L_* is the relative refractive index of the *L*th layer; $H_{n}^{a}$ and $H_{n}^{b}$ are defined in Ref. 39.

1. **The threshold values of *κ*_1_ and *κ*_2_.**


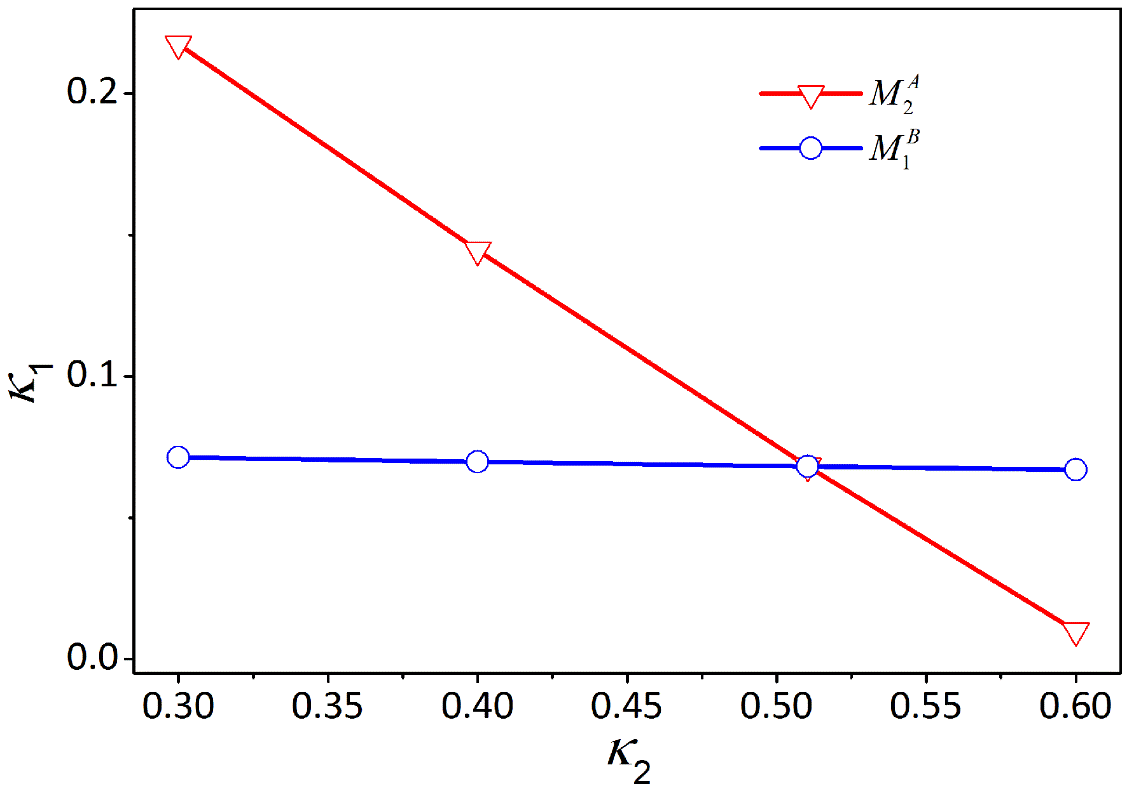


**Figure S3.** The corresponding value of *κ*_1_ and *κ*_2_ at the gain thresholds of $M_{2}^{A}$ and $M_{1}^{B}$ modes. The red curve marked triangle is the $M_{2}^{A}$ mode, and the blue curve marked circle is $M_{1}^{B}$ mode.

The gain coefficients of two gain media are marked as *κ*_1_ and *κ*_2_ respectively. We set *κ*_2_ as a fixed value, $M_{2}^{A}$ and $M_{1}^{B}$ modes can reach to the super-resonance by tuning *κ*_1_. Thus, we obtain the gain threshold value of *κ*_1_. Here we assume that the gain emission covers all the optical frequencies. In Figure S3, there is a cross point at which $M_{2}^{A}$ and $M_{1}^{B}$ modes can reach to the super-resonance simultaneously.

The gain medium encapsulated in the core can be dyes, rare-earth ions (REIs) and quantum dots (QDs). The gain coefficient *κ* as well as the amplification coefficient of light *g* is related to both the emission cross section $\sigma_{e}$ and the concentration *N* of the gain medium, which can be expressed as (ref. 28)

$\kappa=\frac{\lambda}{4\pi}g=\frac{\lambda}{4\pi}N\sigma_{e}$ (S5)

1. **Phase retardation effect tuned by gain medium.**


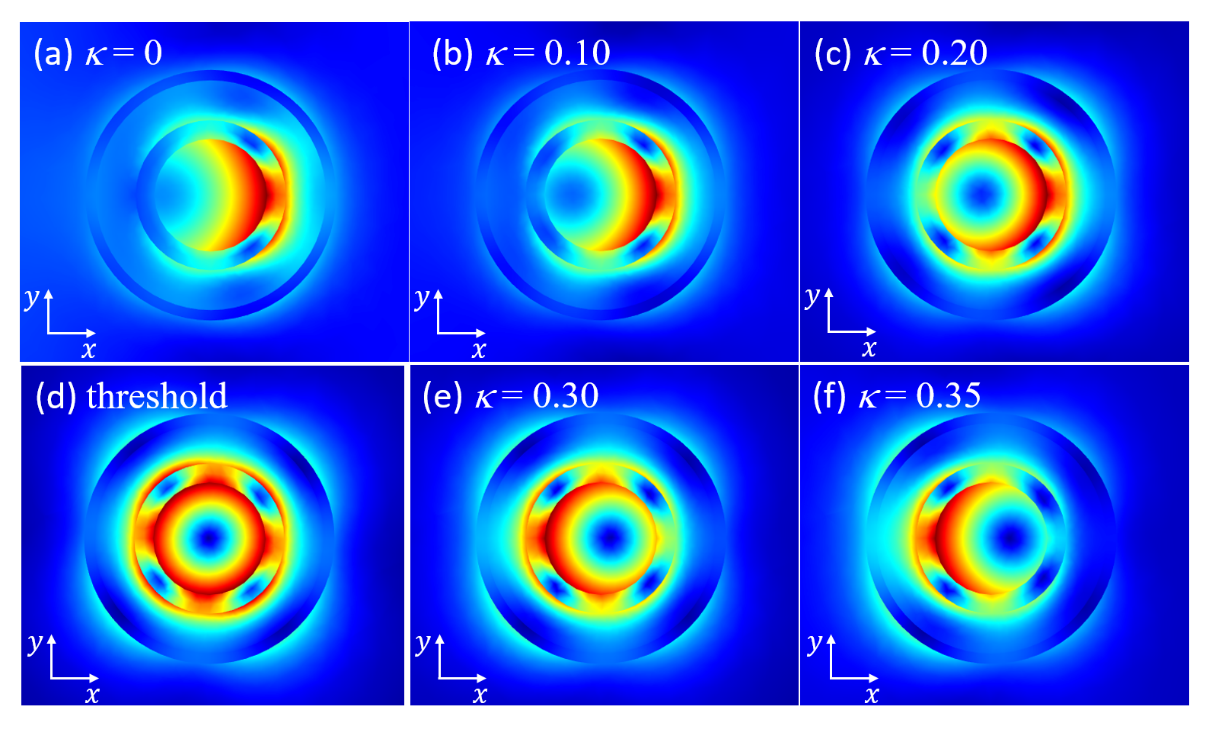


**Figure S4.** Near-field distributions of the $M_{2}^{A}$ mode. The gain coefficient of (a-f) is *κ*_1_ = *κ*_1_ = *κ* = 0, 0.10, 0.20, 0.2454, 0.30 and 0.35 respectively.

Gain media can excite the “pure quadrupole polarization” by modifying the phase. As mentioned in the paper, the quadrupole polarization can be induced by phase retardation effect. However, the retardation effect leads to the asymmetry of near-field. As shown in Figure S4, the near-field distribution is changed in a large extent when gain is introduced into the nanostructure. The retardation effect is found in the passive nanostructure (Figure S4a). When the gain coefficient is increased, the retardation effect is depressed (Figure S4b-S4c). The retardation effect is disappeared completely as the threshold is reached, leading to a symmetrical near-field distribution (Figure S4d). The phase retardation is reversed as the gain coefficient is further increased (Figure S4e-S4f).

1. **Gain** **linewidth.**


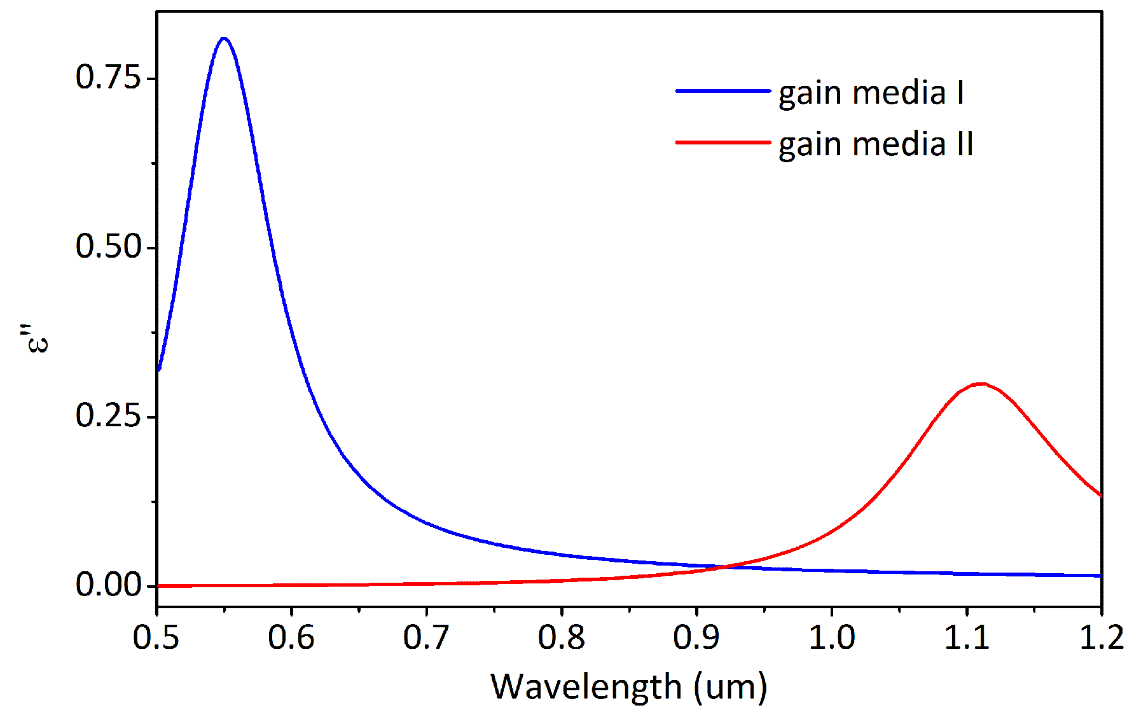


**Figure S5**. The quantum emission spectra of the gain medium. The blue curve is gain media I (Er^3+^) and the red curve is gain media II (IR26).

In experiment, the emission of gain medium is not as large as we assume in the paper. A single Lorentzian emission lineshape of gain medium doped with Er^3+^ ions or IR26 dyes is calculated as $\varepsilon=\varepsilon'-i\varepsilon''=\varepsilon_{b}-\varepsilon''_{g}(\omega_{g})\Delta/[2(\omega-\omega_{g})+i\Delta]$, where $\Delta$ is the emission linewidth, $\varepsilon_{b}$ is the real permittivity of the background medium, $\varepsilon''_{g}(\omega_{g})$ is the maximum value of $\varepsilon''_{g}$, $\omega_{g}$ is the emission central frequency. $\varepsilon_{b}$ of doped Dielectric I and Dielectric II are fixed at 2.045 (silica) and 2.86 (zirconia), respectively. The emission central frequencies of Dielectric I doped with Er^3+^ and Dielectric II doped with (IR26) are at 550 nm and 1110 nm, respectively, and the emission linewidths of Er^3+^ and IR 26 are 0.35 and 0.15, respectively ^a,b^. The quantum emission spectra of the doped Dielectric I and Dielectric II are shown in Figure S5. The SHG calculation results are shown in Figure S6, which is nearly same as than in the paper.


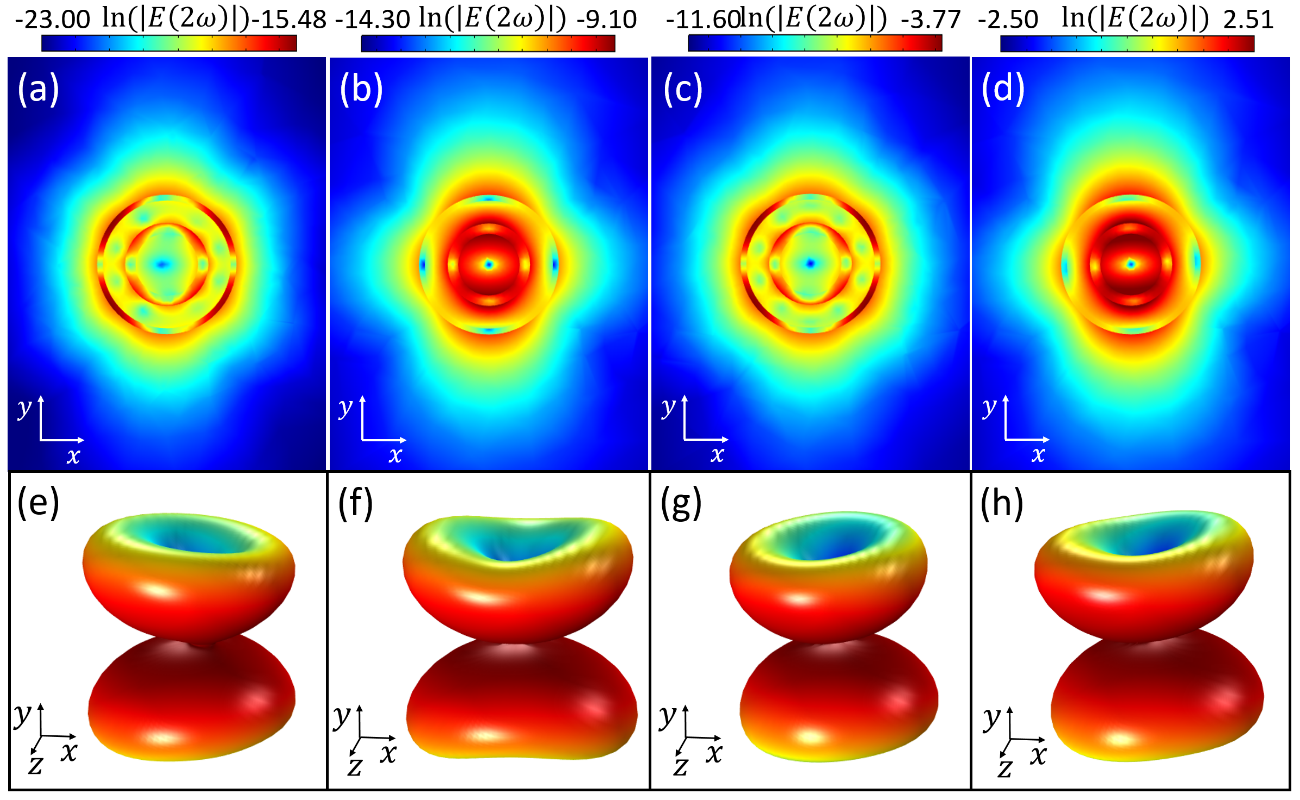


**Figure S6.** Near-field distributions of the SH intensity plotted on logarithmic scale (ln(|*E*(2ω)|) for (a) passive nanostructure, (b) active nanostructure with super-resonance only at mode $M_{2}^{A}$, (c) active nanostructure with super-resonance only at mode $M_{1}^{B}$, (d) active nanostructure with super-resonances at both modes. (e-h) The corresponding 3D far-field distributions in (a-d). All of the SH emissions are quadrupole.

1. **The feature of active nanostructures.**

As shown in Figure S7, the linewidths of the absorption and scattering cross sections are narrowed down as the gain coefficient is increased to the gain threshold, which is getting wide again as the gain coefficient exceed the gain threshold.

When the gain coefficient reaches to the gain threshold, the narrow linewidth of the optical cross sections is very sensitive to the parameters of the nanostructures. We calculate the case that the diameter of silver shell fluctuates from the ideal condition with the deviation of ±1 nm. As shown in Figure S8, the SH near-field intensity divided by the volume shows large enhancement compared with the passive system.


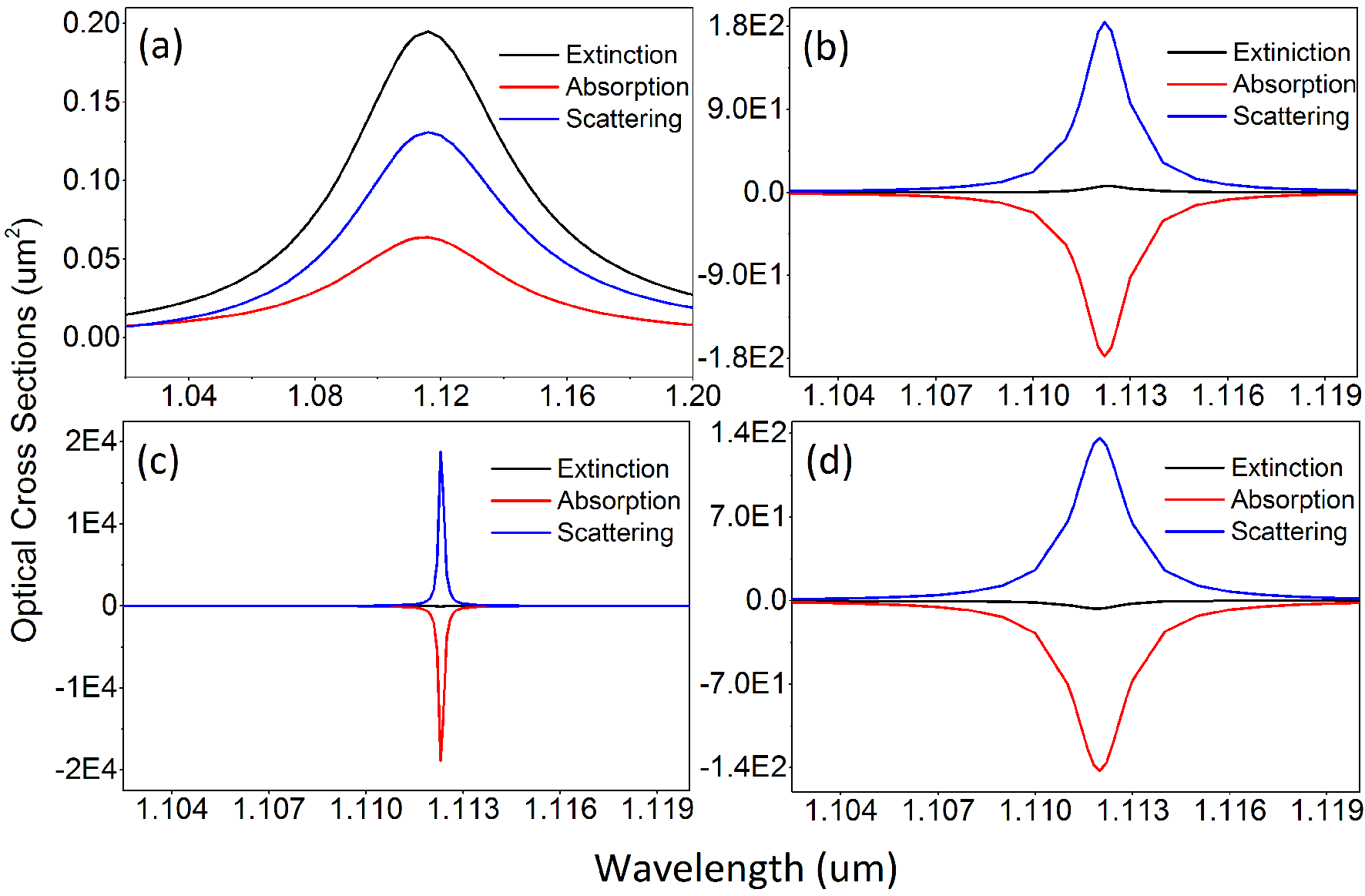


**Figure S7.** Calculated optical cross sections spectra of $M_{1}^{B}$ mode for SDGD nanostructure. (a) Passive nanostructure with *κ*_1_ = *κ*_2_ =0; (b) Active nanostructure with *κ*_1_ = 0.066, *κ*_2_ = 0.49, the gain media are insufficient to compensate the losses of $M_{1}^{B}$mode; (c) Active nanostructure with *κ*_1_ = 0.068, *κ*_2_ = 0.510 (at gain threshold, the losses of $M_{1}^{B}$mode are compensated exactly); (d) Active nanostructure with *κ*_1_ = 0.070, *κ*_2_ = 0.526, the losses of $M_{1}^{B}$mode are overcompensated.


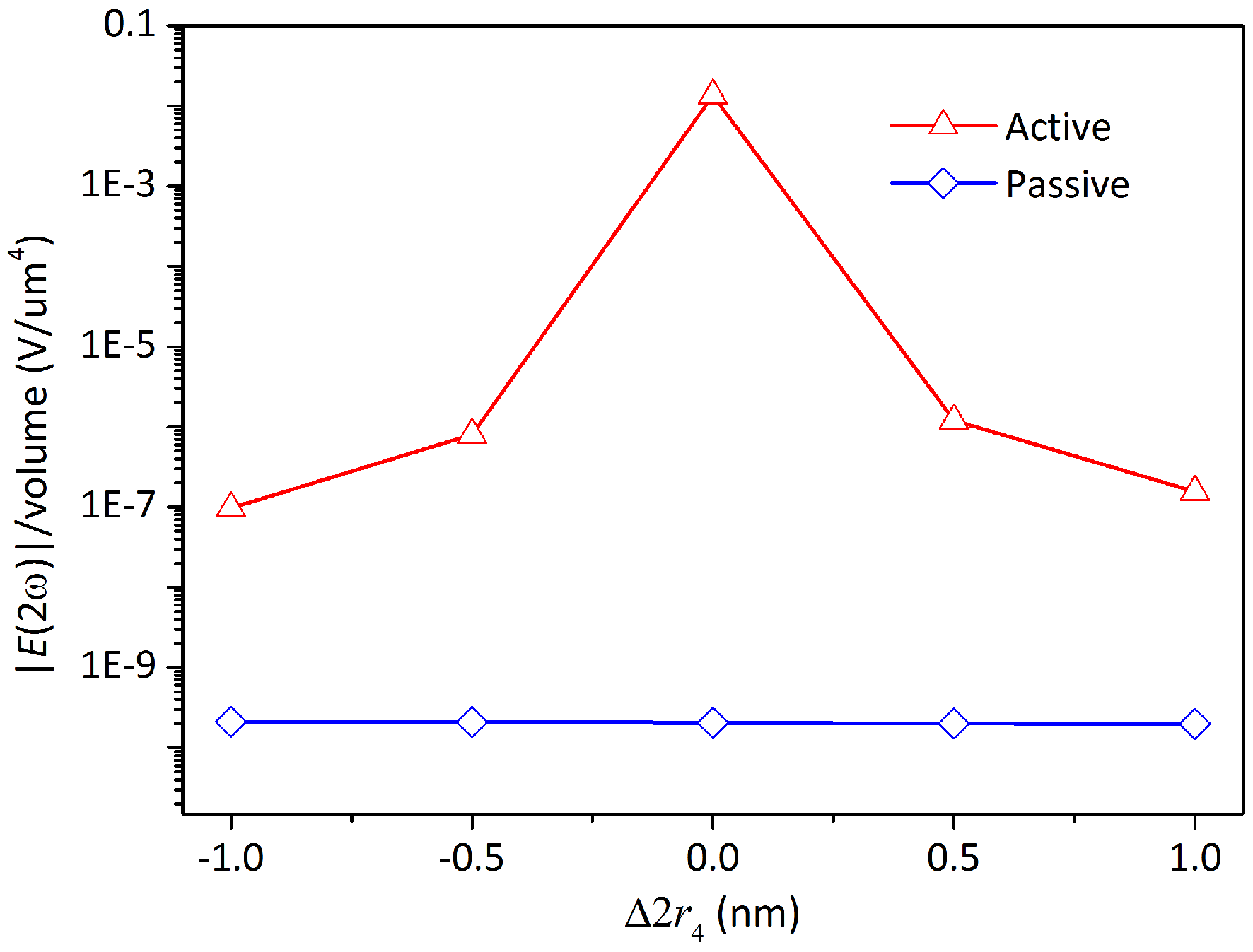


**Figure S8.** SH near-field intensity divided by volume as a function of the deviation of silver shell diameter from the ideal parameters. The red triangles and the blue cubes indicate the active and passive nanostructures, respectively.

**References**

(a) Rivera, V. A. G. Ferri, F. A. Nunes, L. A. O. Zanatta, A. R. Marega Jr., E. Focusing surface plasmon on Er^3+^ ions through gold planar plasmonic lenses. *Appl. Phys. A* **109**, 1037-1041 (2012.)

(b) Hatami, S. Wurth, C. Kaiser, M. Leubner, S. Gabriel, S. Bahrig, L. Lesnyak, V. Pauli, J. Gaponik, N. Eychmuller, A. and Resch-Genger, U. Absolute photoluminescence quantum yields of IR26 and IR-emissive Cd_1-x_Hg_x_Te and PbS quantum dots- method- and material-inherent challenges. *Nanoscale*, **7**, 133-143 (2015).
